# ALA and ALA hexyl ester induction of porphyrins after their systemic administration to tumour bearing mice

**DOI:** 10.1038/sj.bjc.6600559

**Published:** 2002-09-23

**Authors:** C Perotti, A Casas, H Fukuda, P Sacca, A Batlle

**Affiliations:** Centro de Investigaciones sobre Porfirinas y Porfirias (CIPYP), CONICET and Department of Biochemistry, School of Sciences, University of Buenos Aires, Argentine

**Keywords:** photodynamic therapy, PDT, aminolevulinic acid, ALA, ALA derivatives, ALA hexyl ester, blood–brain barrier

## Abstract

The use of synthetic lipophilic molecules derived from 5-aminolevulinic acid (ALA) is currently under investigation to enhance cellular ALA penetration. In this work we studied the effect of systemic administration to mice of the hexyl ester of ALA (He-ALA) on porphyrin tissue synthesis as compared to ALA. In most normal tissues as well as in tumour, He-ALA induced less porphyrin synthesis than ALA after its systemic administration either intravenous or intraperitoneal, although explant organ cultures exposed to either ALA or He-ALA revealed equally active esterases. The only tissue that accumulated higher porphyrin levels from He-ALA (seven times more than ALA) was the brain, and this correlated well with a rapid increase in ALA/He-ALA content in brain after administration of He-ALA. This may be ascribed to a differential permeability to lipophilic substances controlled by the blood–brain barrier, a feature which could be further exploited to treat brain tumours.

*British Journal of Cancer* (2002) **87**, 790–795. doi:10.1038/sj.bjc.6600559 www.bjcancer.com

© 2002 Cancer Research UK

## 

In recent years, 5-aminolevulinic acid-mediated photodynamic therapy (ALA-PDT) has become one of the most promising fields in photodynamic therapy research. ALA is the pro-drug of the photosensitiser Protoporphyrin IX (PpIX). After ALA administration, cells generate PpIX through the haem biosynthetic pathway. The main advantage of PpIX relative to other photosensitisers is the short half life of its photosensitising effects, which do not last longer than 48 h (Kennedy *et al*, 1990; Fukuda *et al*, 1992). Besides, ALA-induced porphyrin fluorescence may also assist in the early detection of some malignancies (Kriegmair *et al*, 1996).

Different approaches are currently under investigation to enhance ALA penetration, such as the application of ALA in various vehicles and the development of new synthetic molecules derived from ALA. Lipophilic derivatives of ALA were expected to have better diffusing properties, and after conversion into the parent ALA by enzymatic hydrolysis, to give a higher PpIX formation rate.

Photodynamic detection and PDT of bladder carcinoma was improved by the use of ALA esters (Marti *et al*, 1999; Lange *et al*, 1999). However, in spite of the success of ALA esters on increasing ALA cell membrane permeation (Kloek and Beijersbergen van Henegouwen, 1996; Kloek *et al*, 1998; Gaullier *et al*, 1997; Berger *et al*, 2000; Uehlinger *et al*, 2000; Casas *et al*, 2001a), the use of these compounds for the treatment of skin cancer is still a matter of discussion, due to the fact that they appear to diffuse slowly across the *stratum corneum* (Casas and Batlle, 2002).

Regarding the systemic administration of ALA and its esterified derivatives, there is some data concerning i.v. and oral administration of ALA (Loh *et al*, 1993; Gil *et al*, 1999), but to the best of our knowledge there is as yet no published data regarding the systemic administration of He-ALA. The aim of this study was, therefore, to study the effect of the systemic administration of He-ALA on porphyrin tissue synthesis as compared to ALA.

## MATERIALS AND METHODS

### Animals

Male BALB/c mice, 12-weeks-old, weighing 20–25 g were used. They were provided with food (Purina 3, Molinos Río de la Plata) and water *ad libitum*. A suspension of 1.65×10^5^ cells of the LM2 cell line (Galli *et al*, 2000) derived from the murine mammary adenocarcinoma M2 (Instituto Roffo, Buenos Aires) was subcutaneously injected into male BALB/c mice. Experiments were performed approximately at day 20 after implantation. Tumours of the same uniform size were employed (1 cm diameter). Animals received human care and were treated in accordance with guidelines established by the Animal Care and Use Committee of the Argentine Association of Specialists in Laboratory Animals (AADEALC), in full accord with the UK Guidelines for the Welfare of Animals in Experimental Neoplasia (UKCCCR, 1988).

### Drugs

ALA was purchased from SIGMA Chem Co., St. Louis, MO, USA. ALA hexyl-ester (He-ALA) was synthesised according to the method previously described by Casas *et al* (1999). All other chemicals were of analytical grade.

### ALA administration

The hydrochloric acid salts of ALA and He-ALA were dissolved in saline in a final volume of 0.15 ml immediately before intraperitoneal (i.p.) injection and in 0.05 ml before intravenous (i.v.) injection.

### Tissue porphyrin extraction

At the indicated times after ALA or He-ALA administration, animals were sacrificed. Before killing, mice were injected with heparin (0.15 ml, 1000 UI) and after sacrifice, they were perfused with 200 ml of sterile saline. The tissue samples were homogenised in a 4 : 1 solution of ethyl acetate : glacial acetic acid mixture. The mixtures were centrifuged for 30 min at 3000 **g**, and the supernatants were treated with an equal volume of 5% HCl. Extraction with HCl was repeated until there was no detectable fluorescence in the organic layer. The aqueous fraction was used for the determination of porphyrins. For fluorometric determination, a Shimadzu RF-510 spectrofluorometer was used, with an emission wavelength of 604 nm and an excitation wavelength of 406 nm, employing PpIX as a reference standard.

### ALA and He-ALA determination in brain

ALA was determined by a modification of the Mauzerall and Granick (1956) method. Five minutes after ALA or He-ALA injection, mice were sacrificed and the brain was rinsed carefully. The tissue was then homogenised in 50 mM Tris HCl buffer pH 7.4 and centrifuged at 3000 **g**. The supernatant was condensed with acetylacetone at 100°C and then centrifuged to precipitate proteins. The ALA or He-ALA content in the resulting supernatant was quantified at 555 nm after addition of the Ehrlich reagent. The nanomoles of ALA and He-ALA per gram of tissue were calculated employing a calibration curve of ALA and He-ALA.

### Organ tissue cultures

The explant tissue culture system developed by Polo *et al* (1988) has been used. Explants were floated in Petri dishes in serum-free minimal essential Eagle's medium (MEM), supplemented with 2 mM L-glutamine and gentamycin (40 μg ml^−1^) and incubated at 37°C in presence of 0.6 mM ALA or He-ALA for 3 h. After ALA or He-ALA exposure, explants of 50 mg were homogenised in a 4 : 1 solution of ethyl acetate-glacial acetic acid mixture and porphyrins extracted and quantified as described above. Optimal conditions for incubations and explant sizes were as determined in a previous work (Fukuda *et al*, 1989).

### Statistical analysis

The unpaired *t*-test was used to establish the significance of differences between groups. Differences were considered statistically significant when *P*<0.05. Three mice per group were employed. *In vitro* experiments were performed three times and run in duplicate.

## RESULTS

### Dose course of porphyrin synthesis after ALA or He-ALA administration via i.p

Figure 1

**Figure 1:**
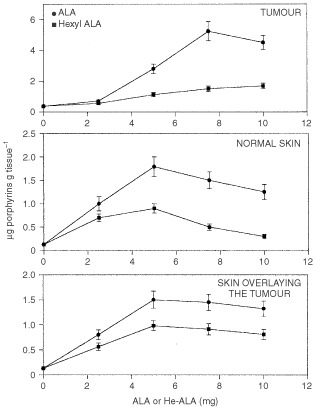
Porphyrin accumulation in tumour, skin and skin overlying the tumour after i.p. administration of increasing ALA or He-ALA doses. Different amounts of ALA or He-ALA were injected i.p. to mice. Three hours later, tissues were excised and porphyrins extracted as detailed in Materials and Methods. Each data point represents the average of three determinations. Error bars show standard deviations.

shows porphyrin synthesis in tumour, normal skin and skin overlaying the tumour (SOT) after the i.p. administration of increasing doses of ALA or He-ALA. In all three tissues, ALA yielded higher levels of porphyrin synthesis than He-ALA.

In tumour tissue 7.5 mg of ALA induced a plateau of 5.23±0.62 μg porphyrin g^−1^ tissue, whereas He-ALA produced only a slight increase of porphyrins in a dose dependant way, reaching a maximal accumulation of 1.68±0.18 μg g^−1^ tissue at 10 mg. He-ALA doses higher than 10 mg are lethal to animals, while ALA doses higher than 30 mg lead to a diminished porphyrin synthesis and, at this dose, the mice autopsy reveled peritoneal thrombus formation.

In normal skin both ALA and He-ALA induced maximal accumulation of porphyrins at a concentration of 5 mg. A similar pattern was observed in the skin overlaying the tumour (SOT) for both compounds reaching plateaux also at 5 mg.

Figure 2

**Figure 2:**
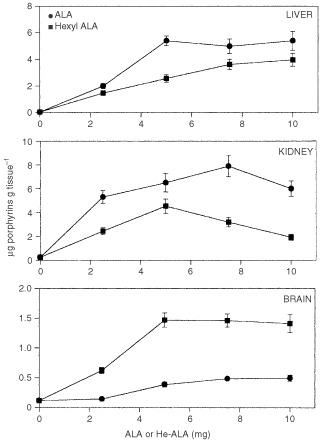
Porphyrin accumulation in liver, kidney and brain after i.p. administration of increasing ALA or He-ALA doses. Different amounts of ALA or He-ALA were injected i.p. to mice. Three hours later, tissues were excised and porphyrins extracted as detailed in Materials and Methods. Each data point represents the average of three determinations. Error bars show standard deviations.

shows liver, kidney and brain porphyrin synthesis after the i.p. administration of ALA or He-ALA. In liver a plateau was observed with 5 mg ALA. A dose dependent increase in porphyrin synthesis was observed with He-ALA, reaching a maximum at 10 mg. In kidney the administration of 7.5 mg ALA induced a maximum amount of porphyrins, while He-ALA produced a slight peak at 5 mg.

The brain was the only tissue where porphyrins induced from He-ALA were higher when compared with ALA. A plateau of 1.47±0.12 μg porphyrins g^−1^ was found with 5 mg of He-ALA, surpassing 50 times basal porphyrin values. Meanwhile, ALA administration reached a plateau of 0.48±0.02 μg g^−1^, only 15 times ground levels.

### Time course of porphyrin synthesis after ALA or He-ALA administration via i.p

Figure 3

**Figure 3:**
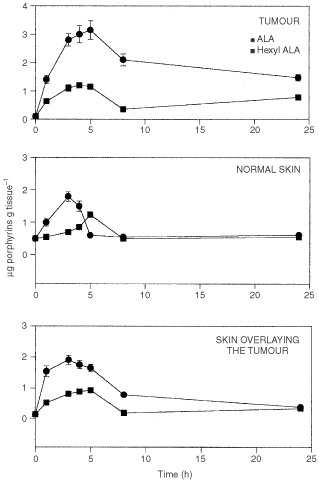
Porphyrin accumulation in tumour, skin and skin overlying the tumour different times after i.p. administration of ALA or He-ALA. 5 mg of of ALA or He-ALA were i.p. injected to mice. At different times, tissues were excised and porphyrins extracted as detailed in Materials and Methods. Each data point represents the average of three determinations. Error bars show standard deviations.

shows porphyrin accumulation for tumour, normal skin and SOT as a function of time after 5 mg of ALA or He-ALA administration via i.p. In these tissues, independently of time, porphyrin accumulation was higher from ALA. In tumour and SOT both ALA and He-ALA induced a peak in porphyrin synthesis between 3 and 5 h. In normal skin ALA produced a peak at 3 h while the peak from He-ALA was at 5 h.

Figure 4

**Figure 4:**
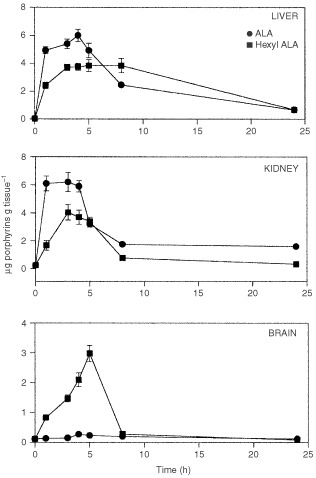
Porphyrin accumulation in liver, kidney and brain at different times after i.p. administration of ALA or He-ALA. 5 mg of of ALA or He-ALA were i.p. injected to mice. At different times, tissues were excised and porphyrins extracted as detailed in Materials and Methods. Each data point represents the average of three determinations. Error bars show standard deviations.

shows porphyrin synthesis in liver, kidney and brain after ALA or He-ALA administration via i.p. In liver, ALA induced a peak at 3 h, and He-ALA, a shoulder between 3 and 8 h and then a decrease in porphyrin synthesis towards basal levels at 24 h after administration. In kidney ALA induced a peak at 3 h and He-ALA a broad peak between 1 and 4 h.

In brain He-ALA produced a maximal porphyrin accumulation of 2.98±0.27 μg g^−1^ at 5 h after administration, while ALA induced a maximum of 0.27±0.03 μg g^−1^ at 4 h. In both cases values return to basal levels 24 h after administration.

Heart, lung, gut, spleen, bladder and ear tissues showed, in general, a porphyrin accumulation from ALA twice higher than that obtained from He-ALA (data not shown).

### Comparison of porphyrin tissue profiles between ALA and He-ALA injected via i.p. and i.v

The profiles of porphyrin accumulation in tumour, skin, SOT, liver, kidney, brain, heart, lung, gut, spleen, bladder and ear after administration of different doses of ALA and He-ALA i.p. and i.v. showed that there were no significant differences between i.p. and i.v. administration for most tissues, independently on the pro-drug used (data not shown).

### Porphyrin synthesis in tissue explants from ALA and He-ALA as an estimation of esterase activities

To determine if the differences on porphyrin accumulation among tissues were due either to ALA/He-ALA uptake and distribution or to differential tissue esterase activities, we performed organ cultures of the different tissues, exposed to either ALA or He-ALA (Table 1

**Table 1:**
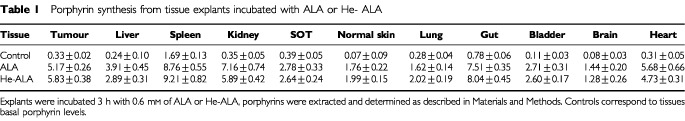
Porphyrin synthesis from tissue explants incubated with ALA or He- ALA

).

We ran tissue explant cultures of tumour, liver, kidney, SOT, normal skin, spleen, lung, gut, bladder, brain and heart. We found that in all these tissues, the amount of porphyrins formed from He-ALA were similar to those obtained from equimolar ALA concentrations, and 10–15 times above basal values.

### ALA and He-ALA accumulation in brain after i.p. administration

Equimolar concentrations of ALA and He-ALA (10 and 15 mg respectively) were injected i.p. to mice. Due to the high toxicity observed for He-ALA leading to rapid death, we sacrificed the animals 5 min after injection. We observed (Table 2

**Table 2:**
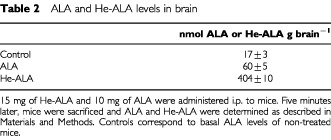
ALA and He-ALA levels in brain

) that 6.7 times more He-ALA is accumulated in brain as compared with ALA.

## DISCUSSION

It has already been demonstrated (van den Akker *et al*, 2000) that after topical application of He-ALA, the *stratum corneum* of the skin acts as a barrier impairing its penetration through the skin. It has also been observed that topical use of He-ALA restricts porphyrin accumulation to the site of application (Casas *et al*, 2001b; Moan *et al*, 2001), but the same intensity of PpIX fluorescence using lower concentrations of ALA esters compared to ALA was obtained in the tissue layers of normal rat colon (Endlicher *et al*, 2001). On the other hand, employing cell lines of different origins such as lung, bladder, glia, breast, lymphoma, leukemia, pancreas and colon among others (Luksiene *et al*, 2001; Casas and Batlle, 2002) porphyrin formation from He-ALA was not impaired, on the contrary it was highly increased by esterification of the ALA molecule.

In this work we have found that porphyrin synthesis from He-ALA is lower than that from ALA in most tissues, and this does not depend on the dose and time after administration of either drug nor on the method of systemic administration (i.v. or i.p.) utilised.

Employing explant organ culture systems, this study has demonstrated, that porphyrin synthesis from He-ALA is equal to porphyrin synthesis from ALA in all tissues. The fact that tissue esterase was equally active in all tissues analysed, indicates that the ester cleavage is not the limiting step on porphyrin synthesis from He-ALA. This also shows that the distribution of either He-ALA, or porphyrins formed from He-ALA, is the reason why porphyrin accumulation is lower when compared with ALA.

On all these grounds, we hypothesise that on the passage of He-ALA from the bloodstream to the tissues, the molecule is retained by vascular structures. The only tissue that accumulates a higher amount of porphyrins from He-ALA is the brain. The zwitterionic structure of aminoacids normally makes the penetration of the lipophilic blood–brain barrier difficult (Krogsgaard-Larsen *et al*, 2000), consequently, it is expected that the use of more lipophilic derivatives of ALA would favour their passage into the brain.

The structure of the blood–brain barrier allows lipophilic molecules such as He-ALA to extravasate and reach the brain, whereas the rest of the capillars retains the molecule. The role of lipophilicity has long been recognised as being important in central nervous system (CNS) penetration by chemicals. Measuring the apparent partition coefficients of the ALA esters between octanol and water (P), it has been demonstrated that the most potent compounds acting on the CNS have a log *P* value of 2±0.5 (Hansch *et al*, 1987). On the other hand, Uehlinger *et al* (2000) determined that the log *P* value for He-ALA was 1.8, whereas log *P* for ALA was −1.5.

Besides having higher lipophilicity, the blood–brain barrier has specific transport systems and carrier proteins (Gloor *et al*, 2001). Although He-ALA transport has not yet been studied in neurons or glial cells, in other cell systems ALA and He-ALA seem to be taken up by distinct mechanisms (Casas and Batlle, 2002; Bermúdez Moretti *et al*, 2002). This may be the reason why delivery of He-ALA to the brain is different from other tissues.

The amount of ALA/He-ALA accumulated in brain 5 min after He-ALA injection is almost seven times higher than the amount found after ALA treatment. This correlates well with the seven-fold increase in brain porphyrin levels from He-ALA as compared with ALA. Since we cannot differentiate ALA from He-ALA with our method of determination, we cannot determine if He-ALA leads to a rapid and sudden death at high concentrations due to a toxicity induced *per se* or if it is rapidly cleaved by brain esterases to release ALA and hexanol, both of them very harmful compounds at high concentrations.

The amount of ALA/He-ALA that we found in brain is equal to 0.5 μM after He-ALA injection and 0.073 μM after ALA injection. The amount of ALA present in CSF of porphyric patients during acute attacks ranges widely between 0.027 and 21 μM (Sweeney *et al*, 1970; Percy and Shanley, 1979; Gorchein and Webber, 1987). However, we cannot predict from these and our values whether or not He-ALA resembles the effects of an acute attack.

In conclusion, we propose that the clinical use of a low dose of He-ALA administered systemically will probably contribute to the delimitation and treatment of brain tumors, although much care should be taken regarding side effects and possible toxicity driven by resulting ALA, He-ALA or its hydrolysis product hexanol.
